# Diversity of Glossinidae (Diptera) species in The Gambia in relation to vegetation

**DOI:** 10.1590/S1984-29612024010

**Published:** 2024-02-19

**Authors:** Alpha Kargbo, Mamudou Jallow, Thallitha Samih Wischral Jayme Vieira, Amien Isaac Amoutchi, Herve Koukoua Koua, Aamir Muse Osman, Rafael Felipe da Costa Vieira

**Affiliations:** 1 WASCAL-Graduate Research Program in Climate Change and Biodiversity, Universite Felix Houphouet-Boigny, Abidjan, Cote d’Ivoire; 2 Department of Physical and Natural Sciences, University of The Gambia, Brikama Campus, The Gambia; 3 Center for Computational Intelligence to Predict Health and Environmental Risks - CIPHER, University of North Carolina at Charlotte, Charlotte, NC, USA; 4 Laboratoire de Zoologie et Biologie Animale, Université de Cocody, Abidjan, Côte d’Ivoire; 5 Laboratório de Doenças Transmitidas por Vetores, Departamento de Medicina Veterinária, Universidade Federal do Paraná - UFPR, Curitiba, PR, Brasil; 6 Somali One Health Centre, Abrar University, Mogadishu, Somalia; 7 Department of Animal Health and Veterinary Services, Ministry of Livestock, Forestry, and Range, Mogadishu, Somalia; 8 Department of Public Health Sciences, University of North Carolina at Charlotte, Charlotte, NC, USA

**Keywords:** Glossina morsitans submorsitans, Glossina palpalis gambiensis, Trypanosomiasis, The Gambia, sub-Saharan Africa, Glossina morsitans submorsitans, Glossina palpalis gambiensis, Tripanosomíases, Gâmbia, África Subsaariana

## Abstract

*Glossina* species are known to transmit African Trypanosomiasis, one of the most important infectious diseases for both livestock and humans in sub-Saharan Africa. Therefore, the aim of this study was to characterize trapped *Glossina* spp. from The Gambia using morphological and molecular techniques in relation to the vegetation cover types. A line transect survey was carried out in all the administrative regions of The Gambia. Tsetse fly trapping was carried out for 14 days during each season using line transect. A total of 220 *Glossina* spp. specimens (117 F and 103 M) were captured, and DNA was extracted from the legs of 100 randomly selected *Glossina* spp. Further, DNA samples were tested by a conventional PCR assay. A total of 135/220 (61%; 95% CI: 54.6-67.8%) and 85/220 (39%; 95% CI: 32.2-45.4%) flies were identified as *Glossina morsitans submorsitans* and *Glossina palpalis gambiensis*, respectively, with most caught during wet season (53.6%) and more females (53.2%) than males. Results of the morphological identification agreed with those of molecular identification. The type of vegetation cover significantly influenced the caught of tsetse flies. Animals and humans at the various trapping sites are at risk of being bitten by tsetse flies.

## Resumo

Espécies de *Glossina* são conhecidas por transmitirem a Tripanossomíase Africana, uma das doenças infecciosas mais importantes para animais de produção e humanos na África subsaariana. Portanto, o objetivo deste estudo foi caracterizar *Glossina* spp. de Gâmbia, usando técnicas morfológicas e moleculares em relação aos tipos de cobertura vegetal. Um transecto linear foi realizado em todas as regiões administrativas de Gâmbia. A coleta de moscas tsé-tsé foi realizada durante 14 dias e em cada estação. Um total de 220 espécimes de *Glossina* (117 F e 103 M) foram capturados e o DNA foi extraído das patas de 100 *Glossina* spp. de forma aleatória. Posteriormente, as amostras de DNA foram testadas por um ensaio de PCR convencional. Um total de 135/220 (61%; 95% CI: 54,6%-67,8%) e 85/220 (39%; 95% CI: 32,2%-45,4%) moscas foram identificadas como *Glossina morsitans submorsitans* e *Glossina palpalis gambiensis*, respectivamente, com mais capturas durante a estação chuvosa (53,6%) e mais fêmeas (53,2%) do que machos. Os resultados da identificação morfológica concordaram com os da identificação molecular. O tipo de cobertura vegetal influenciou significativamente a captura de moscas tsé-tsé. Os animais e humanos nos vários locais de captura correm o risco de serem picados por moscas tsé-tsé.

## Introduction

Tsetse flies are biological vectors of trypanosomes that cause Animal African Trypanosomiasis (AAT) or nagana in cattle, and Human African Trypanosomiasis (HAT) known as sleeping sickness in humans (Losos, 1986). Trypanosomiasis is a substantial productivity-limiting livestock disease in sub-Saharan Africa, contributing to poverty and food insecurity in the region (Holt et al., 2016; Abro et al., 2021).

According to the most recent estimates, 3 million cattle die annually in Africa, costing the continent's Gross Domestic Product (GDP) losses of 4.5 billion US dollars annually (Angara et al., 2014; Oluwafemi et al., 2007; Hassan-Kadle et al., 2019), because of direct (mortality, production losses, costs of prophylactic and curative trypanocidal drugs) and indirect losses due to crop production decline and deficiency of animal protein diets (WOAH, 2013).

HAT is a neglected tropical disease of public health importance. It has been responsible for a considerable degree of suffering within the distributional limits of the vector in sub-Saharan Africa, where it puts about 70 million people at risk and it is generally lethal if left untreated or inadequately treated (Büscher et al., 2017; Franco et al., 2022; Papagni et al., 2023).

Tsetse flies (Diptera: Glossinidae) are large biting and blood-feeding insects comprising 31 species and subspecies from a single genus, *Glossina* (Abd-Alla et al., 2018). *Glossina* species are classified into three groups: palpalis (Nemorhina), morsitans (Glossina), and fusca (Austenina) (Gooding & Krafsur, 2005). All tsetse species can spread trypanosomes, but their relative significance depends on the strength of their contacts with vulnerable hosts (Gooding & Krafsur, 2005; Bouyer et al., 2019). *Glossina morsitans* and *G. palpalis* groups are mainly found in natural savannahs and riverine forest vegetation, respectively. As species of the savannah (Morsitans groups of tsetse) and riverine (Palpalis group) groups occupy different habitats, they have different host preference, use different cues to identify their hosts and are implicated in the transmission of *Trypanosoma* species (Bouyer et al., 2019).

In The Gambia, the Central River (CRR) and the Lower River (LRR) regions are primarily infested with the tsetse fly *G. m. submorsitans,* the primary vector of AAT in the country (Kargbo & Kuye, 2020; Snow et al., 1996). The fly is mainly found in dry, canopied savannah woodland (Kargbo & Kuye, 2020; Snow et al., 1996). *G. palpalis* is also present, although more restricted to riverine vegetation, a region ranked as an area of low to moderate tsetse challenge (Kargbo & Kuye, 2020). In this area, the incidence of trypanosomes in the country has been reported to be higher between October and December (Kargbo et al., 2022a; Olaniyan et al., 2022). A complete understanding of the patterns and factors contributing to *Glossina* distribution can be instrumental in making decisions for better tsetse and trypanosomiasis control measures but requires accurate vector identification. Vector identification is often based on examining genital morphology, wing, and the body color of the flies (Abd-Alla et al., 2018). The hypervariable nature of many of these phenotypic characteristics and body color changes during sample storage may lead to misidentification (Odeniran et al., 2021; Armstrong & Ball, 2005). Although the information on livestock trypanosomiasis in The Gambia has been reported (Rawlings et al., 1993; ITC, 2015; Kargbo et al., 2022a, b), the impact of vegetation on biting flies such as tsetse flies, *Tabanus* and Stomoxys species has for long been studied in many countries in Africa. Many researchers have associated higher infestation of biting flies with the dense vegetation (Rayaisse et al., 2015; Lendzele et al., 2017; Keita et al., 2020; Malele et al., 2011; Lydie et al., 2017). However, the present study differs from other studies in that traps were set in all regions of the country to determine the population prevalence of *Glossina* species, unlike other studies that were based on provincial prevalence, such as in Western Cameroon (Kamdem et al., 2020) and Southern Kaduna Nigeria (Ahmed et al., 2005). Furthermore, the country's climate, landscape, and specific flora along the Gambia River create an environment conducive to various bloodmeal hosts for tsetse flies. Among these factors, the substantial population of warthogs (*Phacochoerus africanus*) throughout the country is noteworthy. This phenomenon is partly attributed to the warthogs being inedible for the majority of the Muslim population (Claxton et al., 1992; Kargbo & Kuye, 2020).

Accordingly, this study aimed to determine the tsetse fauna in selected areas of The Gambia using morphological and molecular techniques to identify the flies, to show their abundance in relation to the vegetation cover type and to further validate the performance of Biconical, NGU and Vavoua traps in catching *Glossina* species in The Gambia.

## Materials and Methods

### Description of trapping sites

**Figure 1:**
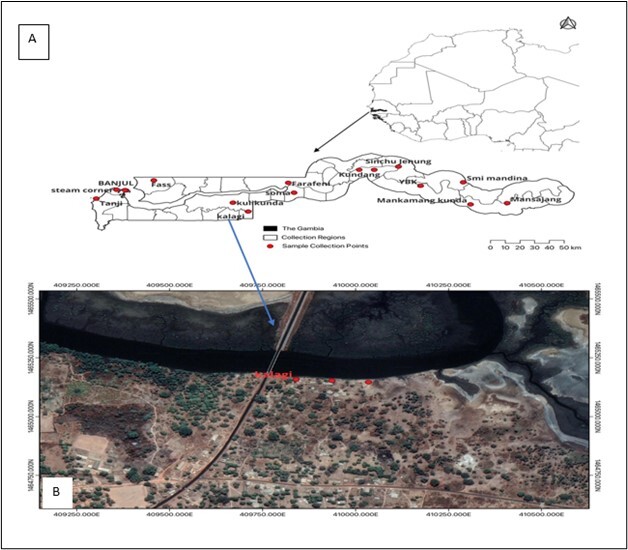
Location of The Gambia in West Africa showing the points where traps were set (A) on a transect of 100 m at village Kalagi in The Gambia (B).

**Figure 2:**
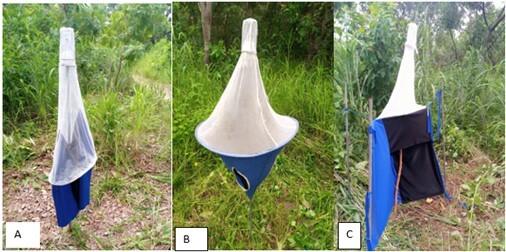
Traps used in seasonal monitoring of tsetse flies: (A) Vavoua trap, (B) Biconical trap, and (C) NGU trap, in The Gambia.

This study was conducted in The Gambia, located in West Africa (Figure 1). Three different traps were set in all the country's regions in October and December 2020 and April and July 2021, based on the wet and dry seasons of the country. A line transect survey was carried out using three traps: the Vavoua, Biconical, and NGU (Kargbo et al., 2021). Traps were set 100 m apart in each of the 11 villages in the rural areas, and five wards in the urban areas were randomly selected for this survey (Figure 1). Figure 1B shows the exact points at which each trap was set in one of the villages. The characteristics at the various trapping sites and the animals at risk of being bitten by tsetse flies are summarized in Table 1. Biconical, NGU, and Vavoua traps were made locally using a regular white mosquito net, phthalogen blue and black fabric (Figure 2). The geographical coordinates for each trapping site were recorded using a Garmin device (GPSMAP GARMIN 65S, United Kingdom). The trapping of tsetse flies took 14 days in each of the following months: October 2020, December 2020, April 2021, and July 2021. The comparative performance of these traps has been previously described (Kargbo et al., 2022b).

### Fly identification and preservation

**Figure 3:**
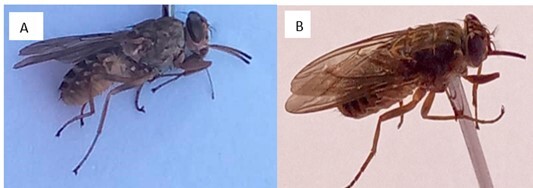
Specimen of the tsetse flies caught: (A) *G. m. submorsitans* and (B) *G. p. gambiensis* in The Gambia.

The morphological identification of tsetse flies was conducted following published morphological identification keys (Service, 2012; Smith, 1973). All specimens were conserved in ethanol and identified using light microscopy (Olympus, Hamburg, Germany). Morphologically, *G. morsitans* and *G. palpalis* were identified (Figure 3), and they were inferred to be *G. m. submorsitans* and *G. p. gambiensis* based on published data (Service, 2012; Smith, 1973). Trapped flies were stored in pools based on the village's catch and seasons. They were kept in 2 mL Eppendorf® Seal-Rite tubes containing 70% ethanol and stored at -20 ^0^C before molecular analysis to authenticate the identification of these specimens.

### Genomic DNA extraction

The flies were placed into 16 pools, with one pool allocated for each species in each region. For the molecular analysis, a random sample was selected, encompassing approximately 30-50% of the specimens from each pool, resulting in a total of 100 specimens *G. m. submorsitans* were obtained from: Upper River Region (URR) = 8, North Bank Region (NBR; n = 5), Banjul (BJL; n = 2), West Coast Region (WCR; n = 3), Kanifing Municipal Council (KMC; n = 10), Lower River Region (LRR; n = 25), Central River Region South (CRR-S; n = 7) and Central River Region North (CRR-N, n = 10), while *G. p. gambiensis* specimens were also from NBR (n = 5), WCR (n = 7), LRR (n = 3), CRR-S (n = 7), CRR-N (n = 10). The preserved flies were taken to the National Public Health Laboratory for DNA extraction using commercial kits (Quick-DNA^TM^ Miniprep Plus Kit, Tustin, California, USA), according to the manufacturer’s instructions. The procedures were as follows: one leg of the fly was crushed in a microcentrifuge tube, and 95 µL of distilled water and solid tissue buffer was added. After, 10 µL of proteinase K was then added. This was mixed thoroughly and then incubated at 55 ^o^C for about 1-3 hours. The content was centrifugated at ≥ 12000 x g for one min. The aqueous supernatant was transferred to a clean tube, and 400 µL of genomic buffer binding buffer was added to the 200 µL supernatant. The mixture was transferred to a Zymo-spin^TM^ IIC-XLR Column in the collection tube. Centrifuge (≥ 12000 x g) for one min. The collection tube with the flow through was discarded. A total of 400 µL DNA pre-wash buffer was added to the column in a new collection tube and centrifuged for 1 min. The collection tube was again emptied, and 700 µL genomic wash buffer was added to the collection tube, which was centrifuged for one minute before emptying it again. Afterward, 200 µL genomic wash buffer was added and centrifuged for one minute. This was discarded with the flow through. It was again transferred to a clean microcentrifuge tube. Finally, ≥ 50 µL of DNA elution buffer was added to the eluate, incubated for 5 min, and then centrifuged for one minute. The DNA concentration ranged between 39 and 90 ng/µL, and the purity index, the ratio of absorbance values obtained at 260 nm and 280 nm (A260/A280), were 1.86-1.97, respectively.

### PCR based identification of tsetse flies

DNA samples were tested by a conventional Polymerase Chain Reaction (PCR) assay targeting a fragment of the ITS1 region, as previously described (Abd-Alla et al., 2018; Bouyer et al., 2006). PCR identification method was carried out as follows. A total of 1.5 uL of the extracted DNA from each sample was placed in a 25 uL PCR reaction tube. The first PCR was conducted using *Glossina* ITS1-specific primers, forward 5ʹ-GTGATCCACCGCTTAGAGTGA-3ʹ, and reverse 5ʹ-GCAAAAGTTGACCGAACTTGA-3ʹ. The following PCR condition was used: 95 °C for 5 min, 30 cycles of 94 °C for 1 min, 62 °C for 1 min, and 72 °C for 90 sec, then 72 °C for 7 min. The second PCR method was used for the *Wolbachia* detection primers as previously described by (Abd-Alla et al., 2018), which were 16S rRNA NI-J-12585 5ʹ-GGTCCCTTACGAATTTGAATATATCCT-3ʹ, and LR-N-12866 5ʹ-ACATGATCTGAGTTCAAACCGG-3ʹ with negative control (no DNA template) and positive DNA samples (DNA of known *Wolbachia*-infected tsetse species). The following PCR program: 94 ^o^C for 5 min, 35 cycles of 94 °C for 45 sec, 55 °C for 45 sec and 72 °C for 30 sec, then 72 °C for 7 min (Dyer et al., 2008). The PCR product was visualized on Agilent TapeStation 4200 capillary electrophoresis instrument.

### Gel electrophoresis

**Figure 4:**
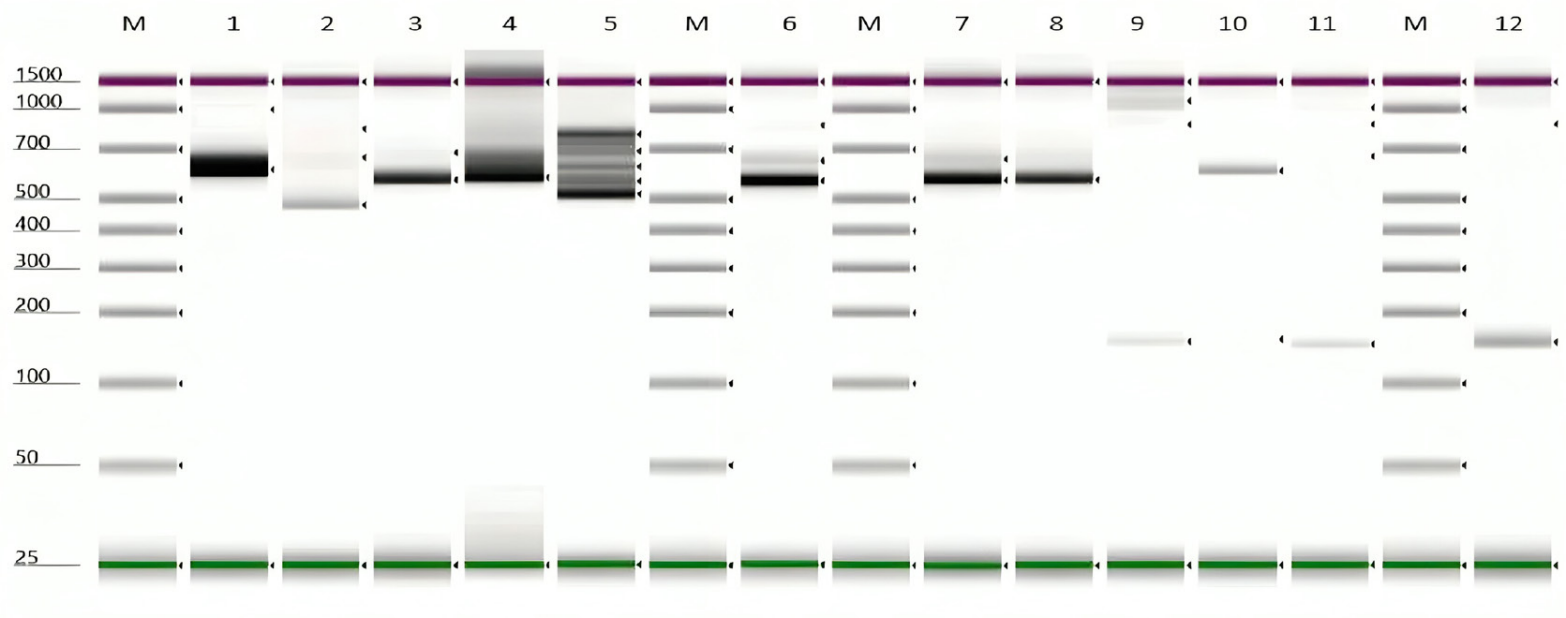
Molecular identification of *Glossina palpalis gambiensis* (543 bp) and *G. m. centralis* or *G. m. submorsitans* (150 bp) caught in The Gambia.

**Figure 5:**
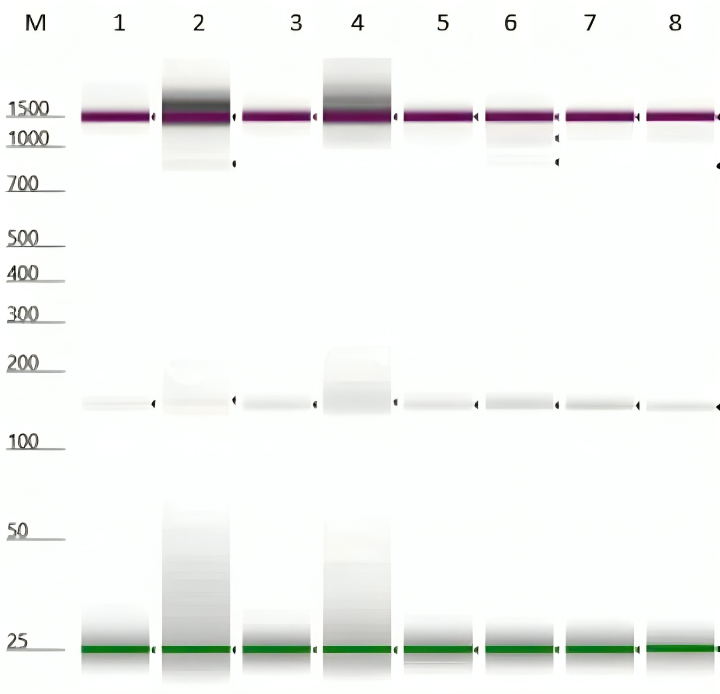
Molecular identification of *G. m. centralis* or *G. m. submorsitans* (150 bp) species caught in The Gambia.

The PCR product was visualized on Agilent TapeStation 4200 capillary electrophoresis instrument. Lane M is the DNA ladder/ marker. The signal size was calculated by comparing its mobility to that of the standards’ bands in the marker lane, as demonstrated by Augustinos et al. (2018). *Glossina palpalis gambiensis* was amplified at 543 bp, while *G. m. centralis* and *G. m. submorsitans* were not amplified using the ITS 1 primer (Figures 4 and 5). However, an additional PCR was conducted to determine the presence/absence of the *Wolbachia*-specific 16S rRNA amplicons for confirming the identification of tsetse species.

### Determination tsetse abundance

The abundance of trapped tsetse was defined as their apparent density (ADT), shown as the amount of tsetse per trap per day (t/t/d), and calculated using the Formula 1 below.


$$ADT=NTC/\left(NT\;x\;NTD\right)$$ (1)

where: ADT: Apparent density; NTC: Number of tsetse flies captured; NT: Number of traps; NTD: Number of trapping days.

### Data collection from vegetation

#### The Normalized Difference Vegetation Index (NDVI) of The Gambia in October 2020

The NDVI index is appropriate technique for detecting the vegetation vigor in a region (Farias et al., 2023). This work unravels different types of land cover in the wet season (2020) in The Gambia through the utilization of NDVI algorithm. Landsat 8 OLI TIRS data was acquired from the 6^th^ to 12^th^, October 2020. Bands 3 and 6 were utilized to generate the NDVI. The images were pre-possessed (mosaic, extracted and masked) with the help of ArcGIS 10.8 software and Erdas Imagine 2015.

#### Normalized Difference Vegetation Index-3rd generation (NDVI3g)

The Normalized Difference Vegetation Index-3^rd^ generation (NDVI3g) using the Global Inventory Monitoring and Modeling System (GIMMS) for Vegetation indices were radiometric measures of photosynthetically active radiation absorbed by chlorophyll in the green leaves of vegetation canopies. The NDVI3g data was downloaded from 2012 to 2021 and it was used in the modeling of the impact of vegetation cover on the flies in The Gambia. This data was obtained for the 10^th^ October, 2020 (NCEI, 2020).

### Data analysis

Collected data were entered into the Microsoft Excel software® and later transferred and analyzed with SPSS Statistics software® (IBM Corp, Armonk, NY, USA, version 26). A Paired sample t-test was used to determine the species and the region caught, differences were considered to be significant when P-values < 0.05. Generalized Addictive Model was used to determine the impact of vegetation cover type on the *Glossina* species flies caught. R version 4.2.1 (R Core Team, 2022) software package was used to carry out the modeling. Generalized Addictive Model was used to determine the impact of NDVI3g on the various flies caught. The models were built to determine the impact of vegetation cover types on *Glossina* species, *Stomoxys calcitrans* and *Atylotus agrestis*. Models were fitted using the observed number of vectors as the outcomes and Generalized Addictive Models (GAM) were used to model the impact of vegetation cover type on the abundance of Glossinidae species, *Stomoxys calcitran* and *Atylotus agrestis*. This model offers a middle ground between simple models, such as linear regression model and more compound machine learning models such as neural networks (Aljoumani et al., 2022). They can be fitted to complex, nonlinear relationships and produce good predictions, while still being able to produce inferential statistics and comprehend and elucidate the underlying model structure. As for the model assessment, only models with least generalized cross validation (GCV) score were chosen. The lower the values of GCV, the better the models fit (Liu et al., 2022).

## Results

### Species diversity and distribution of tsetse flies in The Gambia

A total of 220 *Glossina* spp. specimens (117 F and 103 M) were captured using the NGU (n= 36), Vavoua (n= 41), and Biconical traps (n= 143). The morphological method was used to identify all the 220 specimens caught, while 100 flies were randomly chosen for the molecular analysis. Results of the molecular identification of tsetse flies confirmed those of morphological identification, showing that all trapped tsetse flies belonged to *G. m. submorsitans* and *G. p. gambiensis*.

The seasonal abundance showed that in April 2021, the highest number of *G. m. submorsitans* species (57/135; 34 F and 23 M) were caught, followed by July 2021 (44/135; 26 F and 18 M), October 2020 (22/135; 10 F and 12 M), and December 2020 (12/135; 4 F and 8 M). The highest number of *G. p. gambiensis* was captured in October 2020 (34/85; 14 F and 20 M), followed by July 2021 (18/85; 12 F and 6 M), December 2020 (17/85, 10 F and 7 M), and April 2021 (16/85; 7 F and 9 M). A total of 135/220 (61%; 95% CI: 54.6-67.8%) and 85/220 (39%; 95% CI: 32.2-45.4%) flies were identified as *G. m. submorsitans* (74 F and 61M) and *G. p. gambiensis* (43 F and 42M), respectively. The apparent density of those flies in The Gambia (Table 2) showed that CRR-N (0.19 t/t/d) and LRR (0.18 t/t/d) had the highest density of tsetse flies in this study. *G. m. submorsitans* was caught in all the regions of The Gambia. However, *G. p. gambiensis* was also captured in almost all the regions except in the country's urban rears (Banjul and Kanifing) (Table 2). The highest catches of those flies were from CRR-N, followed by LRR, and the lowest catches were recorded from BJL, with a mean of 28, 26, and 2.5 flies respectively, with no significance difference (Table 3).

### Gel electrophoresis result

The signal/band in lanes 1, 3, 4, 5, 6, 7, 8, and 10 corresponds to 550 bp, which implies that the sample is positive for both *G. p. gambiensis*. The signal band in lanes 9, 11, and 12 correspond to 150 bp, which shows that the specimen was *G. m. submorsitans* (Figure 4). The signal band in lanes 1, 2, 3, 4, 5, 6, 7, and 8 corresponds to 150 bp, which shows that it was *G. m. submorsitans* (Figure 5).

### Impact of vegetation cover and host presence on the distribution of tsetse flies among regions of The Gambia

**Figure 6:**
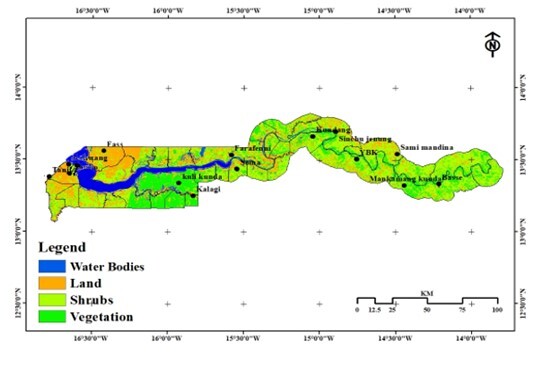
Normalized Difference Vegetation Index (NDVI) of The Gambia in October 2020.

The image from the NDVI algorithm shows four categories: water bodies, land, shrubs, and vegetation (Figure 6). Light green colors denote vegetation, whereas brown colors depict bare ground. The river Gambia and its tributaries can be seen in blue together with thick green coloring that depicts extensive vegetation. In the eastern part of the country, particularly in the Foni area and in the interior, dense wooded land is most prevalent. In the interior of the country as well as in the western Niumi region, there were isolated shrubs. Despite the fact that all the administrative regions in The Gambia had the same number of classifications of vegetation, the WCR (Kalagi) and LRR (Kuli kunda) showed the thickest vegetation, although all other areas also had slightly thick and scattered vegetation. Table 3 shows a significant statistical relationship between fly abundance and type of vegetation cover. Table 4 demonstrates how the trapping locations were perfect niches and environments for capturing these insects. It also has a good probability of at least finding a suitable host for the *Glossina* species, since it has the correct vegetation that they require to survive.

### Predicting the relative abundance of flies using NDVI3g from 2012 to 2021

**Figure 7:**
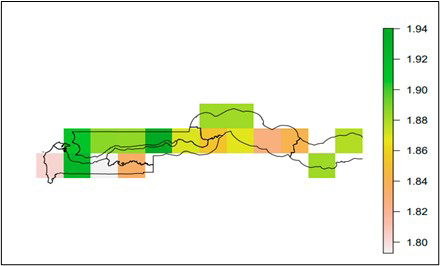
Predictive map of *G. m. submorsitans* abundance in The Gambia.

**Figure 8:**
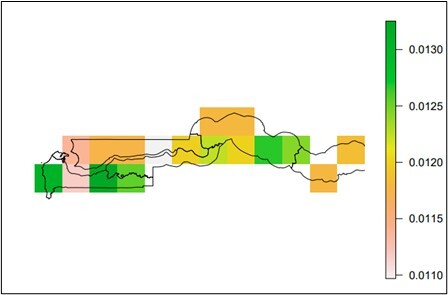
Predictive map of *G. p. gambiensis* abundance in The Gambia.

The results from the predictive mapping of *Glossina* spp. in Figure 7 and 8 shows the predicted values ranged from 0.011 to 1.94. The hotspots areas for *G. m. submorsitan* were located WCR, NBR, URR and some parts of LRR of The Gambia (Figure 7). The hotspots area for *G. p. gambiensis* were in WCR and the western part of CRR-N and CRR-S of The Gambia (Figure 8).

## Discussion

The aim of this study was to use both morphological and molecular methods to identify tsetse flies in Gambia. During the current entomological survey conducted in 16 communities, 220 Glossina spp. were captured. Results of the morphological identification of tsetse flies agreed with those of molecular identification, showing that all the caught tsetse flies belonged to *G. m. submorsitans* and *G. p. gambiensis*. The data showed that *G. m. submorsitansis* widely distributed in all regions of Gambia. *G. palpalis gambiensis* was primarily found in areas close to rivers as it prefers dense evergreen vegetation, heavier shade, and humid habitats near riverine thickets.

Most flies (53.6%) were trapped in July and October, which represents the wet season in The Gambia, compared with low catches observed in April and December (46.4%), which represent the dry season in the country, as reported in previous studies from Nigeria (Orji et al., 2015; Ahmed et al., 2005). The scarce of *Glossina* spp. during the dry season may be characterized by environmental factors unfavorable to tsetse flies' ability to reproduce, grow, and survive, such as dry soil, high temperatures, and low humidity (Pagabeleguem et al., 2016).

The majority of the captured flies were females (53.2%), as reported in previous studies in Nigeria and Cameroon (Orji et al., 2015; Kamdem et al., 2020), and this might be explained by trapping bias, in addition to the common movements of females and males for blood meals, females also search for places to deposit their larvae.

In the present study, regional variations in the distribution of *Glossina* species were observed. *G. m. submorsitans* was the most prevalent in LRR (47), followed by CRR-N (20), while *G. p. gambiensis* in CRR-N (36) and CRR-S (17) showed the highest capture rates, respectively. Moreover, *G. p. gambiensis* were not caught in URR, KMC, and BJL. This difference may be influenced by underlying regional climatic parameters, rainfall, temperature, humidity, wind speed, and vegetation type. Additionally, a previous study has failed on finding *G. m. submorsitans* in the southern region of the country (Kargbo & Kuye 2020; Rawlings et al., 1993). Changes in the climate conditions of those settlements may have driven the spread of *G. m. submorsitan* to the southern region of The Gambia.

Findings obtained herein are consistent with those of Rawlings et al. (1993), who reported *G. p. gambiensis* in evergreen forests and woodlands near the shore and riparian habitats along the river Gambia and its main tributaries. The general rule is that tsetse densities decrease as one moves away from the water. However, Banjul and Kanifing municipal council areas had riverine vegetation, but no *G. p. gambiensis* was caught. This fact may be due to the lack of enough livestock activities, the saltiness of the river water, or the level of urbanization in those areas.

Even though this disease is underreported in the country, as noted by Kargbo et al. (2022b), the consequence of the presence of tsetse flies in all of The Gambia's rural areas provides higher hazard to the rural populace. These results are consistent with the World Health Organization (WHO) reports (WHO, 2023). They explained that tsetse flies expose humans who lived in rural regions to HAT, and Cecchi et al. (2015) stated that tsetse flies may represent veterinary and medical risks.

Mapping entomological data enabled the specification of suitable places for an effective control program. In this light, vector control in The Gambia should be focused on the forest, around water points, along the rivers, and farmlands, because of livestock and wildlife activities and trypanosome infection rates are higher within and around these biotopes.

It is acknowledged that this study has certain limitations, including the small sample size obtained during fieldwork and the inability to obtain high-quality DNA sequencing for phylogenetic analysis. Additionally, it is important to note that PCR was not performed with blood meals of *Glossina* spp. for *Trypanosoma* spp., which could have provided valuable insights into the transmission dynamics of this protozoa. Despite these limitations, the present study provides insights into the prevention and control measures for trypanosomiasis and lays the foundation for further research in this field.

## Conclusions

This study revealed the presence of *G. m. submorsitans* and *G. p. gambiensis* in The Gambia. To the best of the author’s knowledge, this is the first report of *G. m. submorsitans* in the southern part the country. Their presence may pose a significant risk to animals and public health. Further studies on transmission patterns of *Glossina* spp. to livestock and humans will be necessary to comprehend the epidemiology and management of AAT and HAT in the country.

## Supplementary Material

Supplementary material accompanies this paper.

Table 1 Summary table of the description of the trapping sites

Table 2 Distribution of tsetse flies in The Gambia

Table 3 A Paired sample t-test analysis of tsetse fly abundance in regions of The Gambia.

Table 4 The impact of vegetation cover on the abundance of tsetse flies in The Gambia.

This material is available as part of the online article from https://doi.org/10.1590/S1984-29612024010
