# Supplementary material for: Diversity of Glossinidae (Diptera) species in The Gambia in relation to vegetation
Source: Rev Bras Parasitol Vet. 2024 Feb 19;33(1):e012623. doi: 10.1590/S1984-29612024010 (PMC10927271; doi:10.1590/S1984-29612024010)
Supplement: Table 1 [file rbpv-33-1-e012623-s01.pdf]

Diversity of Glossinidae (Diptera) species in The Gambia in relation to vegetation

Diversidade de espécies de Glossinidae (Diptera) em Gâmbia em relação à vegetação

Alpha Kargbo[1, 2]*; Mamudou Jallow[2]; Thallitha Samih Wischral Jayme Vieira[3]; Amien Isaac Amoutchi[1]; Herve Koukoua Koua[4]; Aamir Muse Osman[3, 5, 6]; Rafael Felipe da Costa Vieira[7, 8]*

[1]WASCAL-Graduate Research Program in Climate Change and Biodiversity, Universite Felix Houphouet-Boigny, BP V34, Abidjan, Cote d'Ivoire

[2]Department of Physical and Natural Sciences, University of The Gambia, Brikama Campus, P. O Box 3530, Serrekunda, The Gambia

[3]Laboratório de Doenças Transmitidas por Vetores, Departamento de Medicina Veterinária, Universidade Federal do Paraná - UFPR, Curitiba, PR, Brasil

[4]Laboratoire de Zoologie et Biologie Animale, Université de Cocody, 22 BP 582 Abidjan 22, Côte d'Ivoire

[5]Somali One Health Centre, Abrar University, Mogadishu, Somalia

[6] Department of Animal Health and Veterinary Services, Ministry of Livestock, Forestry, and Range, Mogadishu, Somalia

[7]Department of Public Health Sciences, University of North Carolina at Charlotte, Charlotte, USA.

[8]Center for Computational Intelligence to Predict Health and Environmental Risks (CIPHER), University of North Carolina at Charlotte, Charlotte, USA

How to cite: Kargbo A, Jallow M, Vieira TSWJ, Amoutchi AI, Koua HK, Osman AM, et al. Diversity of Glossinidae (Diptera) species in The Gambia in relation to vegetation. *Braz J Vet Parasitol* 2024; 33(1): e012623. https://doi.org/10.1590/S1984-29612024010

Received August 27, 2023. Accepted December 19, 2023

***Corresponding author: Alpha Kargbo.** E-mail: akargbo@utg.edu.gm; Rafael Felipe da Costa Vieira. E-mail: rvieira@charlotte.edu

Table 1. Summary table of the description of the trapping sites

S/N	Region	Trapping Sites	Characteristics of sites	Trapping period
1.	WCR	Tanji bird reserve	Open gallery forest, game river, riverbanks, tourist and livestock grazing sites	Traps were set from 8 am to empty at 6 pm<br>Traps were set from 7 am to empty at 6 pm
		Kalagi	Riverbank, mangrove vegetation, livestock drinking point	
2.	BJL	Bond road	Open savanna land, giant lizards, fishes, frogs, snakes	Traps were set from 8 am to empty at 6 pm
		Mile two	Riverbank, mangrove forest, prisoners, prison workers	
3.	KMC	Jeswang	Open mangrove savanna, vegetable gardeners	Traps were set from 8 am to empty at 6 pm
		Abuko	Protected forest area, wild animals, livestock market	
4.	NBR	Farafenni	Farmland near riverbank rice growers	Traps were set from 6 am to empty at 6 pm
		Fass	Open savannah	
5.	LRR	Kuli Kunda	Open savannah, livestock drinking, and grazing area	Traps were set from 6:30 am to empty at 6 pm
		Soma	Close to river, savannah, rice growers, and cattle farmers	
6.	CRR-S	Kudang	Open savanna, livestock drinking, and grazing points, wild animal (warthog)	Traps were set from 6 am to empty at 6 pm
		YBK	Gallery forest, livestock grazing area	

7.	CRR-N	Sami mandina	Farmland, gazing area for livestock animals	Traps were set from 6 am to empty at 5 pm
		Sinchu jenung	Open savanna, riverbank, grazing and drinking areas for livestock animals, wild animals (warthog, hippotamus)	
8.	URR	Mankamang Kunda	Open savannah, livestock grazing areas.	Traps were set from 6 am to empty at 5 pm
		Basse (Mansanjang)	Mangrove gallery forest, monkeys, snakes	

Note: LRR (Lower River Region), CRR-S (Central River Region- South), CRR-N (Central River Region-North, URR (Upper River Region), WCR (West Coast Region), NBR (North Bank Region), KMC (Kanifing Municipal council), BJL (Banjul).

Table 2. Distribution of tsetse flies in The Gambia

Species	LRR	CRR-N	CRR-S	URR	WCD	NBR	KMC	BJL	Traps type used			Total
									Biconical	NGU	Vavoua	
G. m. submorsitans	47	20	14	16	5	9	19	5	92	24	19	135
G. p. gambiensis	6	36	17	0	13	13	0	0	51	12	22	85
Total	53	56	31	16	18	22	19	5	143	36	41	220
Percentages	(24%)	(26%)	(14%)	(7%)	(8%)	(10%)	(9%)	(2%)				
TAD	0.158	0.167	0.092	0.048	0.054	0.065	0.057	0.015				

LRR (Lower River Region), CRR-N (Central River Region-North), CRR-S (Central River Region- South), URR (Upper River Region), WCD (West Coast Region), NBR (North Bank Region), KMC (Kanifing Municipal Council), BJL (Banjul) and TAD (Trap Apparent Density)

Table 3. A Paired sample t-test analysis of tsetse fly abundance in regions of The Gambia.

One-Sample Test

	T	Df	*P value*	Mean Difference	95% Confidence Interval of the Difference	
					Lower	Upper
LRR	1.293	1	0.419	26.500	-233.98	286.98
CRR-N	3.500	1	0.177	28.000	-73.65	129.65
CRR-S	10.333	1	0.061	15.500	-3.56	34.56
URR	1.000	1	0.500	8.000	-93.65	109.65
WCD	2.250	1	0.266	9.000	-41.82	59.82
NBR	5.500	1	0.114	11.000	-14.41	36.41

KMC	1.000	1	0.500	9.500	-111.21	130.21
BJL	1.000	1	0.500	2.500	-29.27	34.27

Table 4. The impact of vegetation cover on the abundance of tsetse flies in The Gambia.

**Variable**	***G. m. submorsitans***		***G. p. gambiensis***	
	Estimate	P value	Estimate	P value
**Farmland**	-466e-01	<0.001*	-1.1994	<0.001
**Gallery forest**	0.915	0.016*	0.0684	0.016
**Mangrove forest**	1.000	0.087	0.560	0.003
**Summary of model terms**	R^2=0.509		R2=0.402	
	DE=10.1%		DE=13.2%	
	GCV=0.559		GCV=0.690	

Note: * statistically significant
